# Angiotensin-Converting Enzyme Inhibitors and Metabolic Aging: A Drosophila Perspective

**DOI:** 10.3390/biom15101378

**Published:** 2025-09-28

**Authors:** Denise Vecchie’, Victoria G. Faber, Patricia Jumbo-Lucioni, Robert R. H. Anholt, Trudy F. C. Mackay, Maria De Luca

**Affiliations:** 1 Department of Nutrition Sciences, University of Alabama at Birmingham, Birmingham, AL 35294, USA; dvecchie@uab.edu (D.V.); vgfaber@uab.edu (V.G.F.); 2 Department of Pharmaceutical, Social and Administrative Sciences, Samford University, Birmingham, AL 35229, USA; pjumbolu@samford.edu; 3 Department of Genetics and Biochemistry and Center for Human Genetics, Clemson University, Greenwood, SC 29646, USA; ranholt@clemson.edu (R.R.H.A.); tmackay@clemson.edu (T.F.C.M.)

**Keywords:** aging, metabolism, Drosophila, renin-angiotensin system blockade

## Abstract

Aging is characterized by a progressive decline in physiological function that impairs performance and increases vulnerability to disease and mortality. Delaying this deterioration is key to promoting healthy aging. Age-associated functional decline is closely linked to alterations in intermediary metabolism, including disrupted lipid metabolism and impaired mitochondrial function. Counteracting these metabolic changes, particularly those affecting basal metabolic rate and energy utilization, may be a feasible strategy to extend healthspan. The Renin-Angiotensin System (RAS), which controls blood pressure through Angiotensin II, an octapeptide hormone generated from Angiotensin I by Angiotensin-Converting Enzyme (ACE), has been identified as a potential target for aging therapies. ACE inhibitors, such as the commonly prescribed vasodilator lisinopril, have been shown to exert beneficial effects on healthspan. Disentangling their systemic effects from direct cellular actions on intermediary metabolism is challenging in humans but can be pursued in model organisms. *Drosophila melanogaster* expresses two ortholog of mammalian ACE, Ance and Acer, which have diverged to acquire different functions. Since fundamental cellular processes are evolutionarily conserved and flies have an open circulatory system, Drosophila provides a versatile model for translational studies on ACE inhibition and aging. Recent studies in Drosophila reveal sex-, age-, and genetic background-specific effects of lisinopril on metabolic rates and aging-related organismal phenotypes. Integrating preclinical findings from Drosophila with clinical studies will be essential to define the therapeutic potential of RAS inhibition in extending lifespan and delaying aging.

## 1. Introduction

Population aging poses considerable economic and social challenges for both governments and families [1]. In light of these challenges, scientists have made significant efforts to understand the biological underpinnings of healthy aging [2] and the evolutionary forces that have shaped the aging process across species [3].

Aging (or senescence) is characterized by a progressive decline in physiological functions that compromises an organism’s ability to survive and/or reproduce with advancing age [3]. There are three prevalent evolutionary genetic theories to explain the aging process: mutation accumulation, antagonistic pleiotropy, and the disposable soma theory. All are based on the fundamental assumption that the force of natural selection, which favors traits that enhance early-life fitness, declines with age, thereby allowing biological damage to accumulate (reviewed in [3,4]). Specifically, the mutation accumulation theory suggests that aging results from the buildup of late-acting deleterious mutations [5], the antagonistic pleiotropy theory posits that some genes have beneficial effects early in life but harmful effects in later life [6], and the disposable soma theory argues that organisms allocate limited resources to reproduction at the expense of somatic maintenance [7]. Supported by extensive empirical evidence [3,4], these theories have provided compelling explanations for why aging has persisted across species and how longevity has evolved [8].

In parallel to evolutionary theories of aging, molecular studies conducted over the past three decades in both humans and model systems have revealed key mechanisms underlying aging and longevity [9,10]. This body of work has firmly established that the potential to promote lifespan extension is rooted in interconnected, evolutionarily conserved hallmarks of aging, including genome instability, epigenetic alterations, telomere attrition, and loss of proteome homeostasis, as well as mitochondrial dysfunction, dysregulated nutrient sensing, chronic inflammation, and cellular senescence [11]. The identification of these cellular and molecular hallmarks of aging has underscored the potential for both lifestyle modifications and pharmacological interventions to promote healthy aging and longevity [12], and has paved the way for the emergence of the interdisciplinary field of geroscience [13]. The central tenet of geroscience is that aging is the main risk factor for most non-communicable diseases, including type 2 diabetes (T2D), cardiovascular disease, chronic obstructive pulmonary disease, and Alzheimer’s disease [14], which are among the leading causes of disability and mortality worldwide [15]. As such, a pressing objective in gerontology is to pursue investigations aimed at extending healthspan alongside lifespan to achieve optimal longevity [16,17] or, alternatively, to increase average life expectancy while compressing morbidity into a brief period before death [18].

Several longevity-enhancing interventions have been identified to also enhance healthspan in model organisms [19]. These include dietary interventions, modulation of nutrient-sensing pathways, senolytic therapies that target non-dividing senescent cells, mitochondrial-targeted compounds, anti-inflammatory agents, and epigenetic modulators [19]. Several of these strategies have been systematically evaluated by the U.S. National Institute on Aging through the Interventions Testing Program (ITP) and the *Caenorhabditis* ITP, which have generated robust and reproducible data supporting their potential to enhance both lifespan and healthspan [20,21,22]. However, not all these interventions compress morbidity. Recent theoretical evidence derived from studies in invertebrates and mice suggests that longevity interventions that steepen the survival curve are more likely to compress morbidity [23]. In contrast, interventions that extend lifespan by proportionally shifting the survival curve may increase healthspan without compressing morbidity. This work revealed that, among all interventions tested by the ITP, senolytics, the anti-diabetic acarbose 17α-estradiol, the ketone body precursor 1,3-butanediol, and the Angiotensin-Converting Enzyme (ACE) inhibitor captopril are predicted to both extend lifespan and compress morbidity [23]. ACE inhibitors, such as captopril, lisinopril, enalapril, and perindopril, function by blocking the activity of ACE, a key component of the Renin-Angiotensin System (RAS). The RAS has emerged as an important modulator of aging and healthspan (reviewed in [24,25]) and the identification of captopril as one of the predictive interventions that also compress morbidity further supports the potential of ACE inhibitors as promising candidates for research into healthy aging and lifespan extension.

Metabolic aging is a central driver of physiological decline across tissues and organ systems. This process is characterized by dysregulation of key pathways involved in energy production, redox balance, and nutrient sensing, including insulin/insulin-like growth factor-1, AMP-activated protein kinase, and mechanistic targets of rapamycin signaling [26]. Clinical and preclinical studies in rodent models have demonstrated that pharmacological inhibition of the RAS can improve age-related metabolic dysfunction by reducing oxidative stress and inflammation, enhancing mitochondrial efficiency, and improving insulin sensitivity and tissue perfusion [24,25]. These benefits are attributed to both systemic and tissue-specific actions.

The RAS was first identified as a critical regulator of mammalian blood pressure in the 1950s [27]. However, in mammals, it remains challenging to disentangle the vascular effects of RAS inhibitors from their cell-intrinsic mechanisms. Components of the RAS are evolutionarily conserved and have been detected in non-mammalian organisms lacking closed circulatory systems, such as *D. melanogaster* [28]. *D. melanogaster* possesses an open circulatory system and can serve as a model for uncovering the molecular mechanisms through which RAS blockade supports metabolic homeostasis with age. Additionally, due to its short lifespan and the richness of available genomic tools, *D. melanogaster* enables the exploration of trade-offs predicted by evolutionary theories of aging by allowing researchers to examine how pharmacological inhibition of the RAS may differentially affect reproduction, stress resistance, or metabolic efficiency across diverse genetic backgrounds. Importantly, about 75% of human disease-associated genes have Drosophila orthologs and fundamental metabolic processes are evolutionary conserved [29].

In this review, we summarize emerging evidence from *D. melanogaster* showing that ACE inhibitors influence metabolic aging, and we highlight the potential of this model to uncover novel mechanisms that may inform therapeutic strategies that target RAS for metabolic and age-related diseases in humans.

## 2. RAS: Canonical and Non-Canonical Pathways

The RAS is an essential endocrine and paracrine system that regulates blood pressure, fluid balance, and electrolyte homeostasis [30]. The canonical pathway begins when renin, a highly specific protease released by the kidney in response to low blood pressure or sodium levels, cleaves angiotensinogen, a substrate of hepatic origin, to form the inactive decapeptide Angiotensin I (Ang I). Ang I is then converted into the active octapeptide Ang II by ACE, a metalloprotease predominantly secreted by the lungs [31]. The actions of Ang II, the primary effector molecule, are mediated through its binding, with equal affinity, to two main G protein-coupled receptor subtypes: type 1 receptor (AT1R) and type 2 receptor (AT2R). AT1R accounts for most of the classical physiological actions of Ang II, such as vasoconstriction, aldosterone release, inflammation, ion transport, cellular growth, and migration [32]. These receptors are predominantly found in the vascular smooth muscle, heart, kidneys, and brain. Upon Ang II binding, AT1R undergoes conformational changes that activate downstream signaling cascades through G proteins, leading to the stimulation of phospholipases, nicotinamide adenine dinucleotide/nicotinamide adenine dinucleotide phosphate (NADH/NADPH) oxidases, adenylyl cyclase, protein kinase C isoforms, and ion channels, including L-type and T-type calcium channels [32]. In contrast, AT2Rs are mostly expressed in fetal tissues. Their expression decreases in adulthood, persisting mainly in the heart, kidneys, adrenal glands, and brain [33]. Although less well understood, AT2Rs generally counterbalance AT1Rs effects by promoting vasodilation, cell growth inhibition, anti-proliferative, and pro-apoptotic effects through several signaling cascades [33].

**Figure 1:**
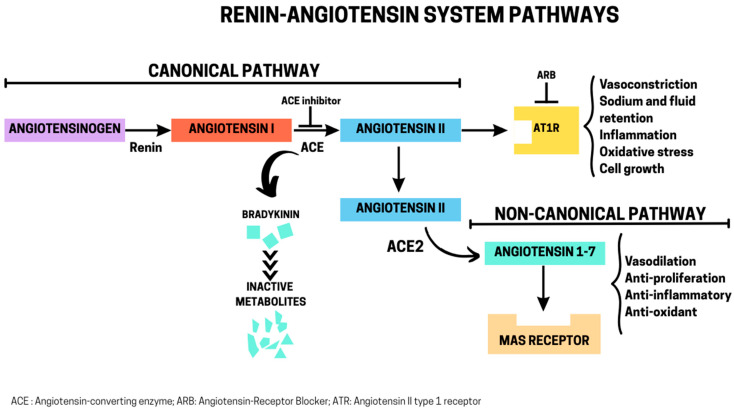
Canonical and non-canonical renin–angiotensin system (RAS) pathways in humans. Renin cleaves angiotensinogen to angiotensin I, which is converted by ACE to angiotensin II in the canonical pathway. Ang II binding to AT1R mediates vasoconstriction, aldosterone release, inflammation, and cell growth. ACE inhibitors block the conversion of angiotensin I to angiotensin II and prevent the degradation of bradykinin. Angiotensin receptor blockers prevent Ang II from binding AT1R. In the non-canonical axis, ACE2 converts angiotensin II to Ang (1-7), which signals via Mas to drive vasodilation, anti-inflammatory, and anti-proliferative activities. The dynamic balance between these axes is critical not only for blood pressure control but also for influencing aging trajectories, metabolic health, and tissue resilience.

An alternative non-canonical pathway is the ACE2/Ang (1–7)/Mas receptor axis, in which ACE2 converts Ang II into the heptapeptide Ang (1–7). Ang (1–7) then binds to the Mas receptor, another G protein-coupled receptor, as well as AT2Rs (extensively reviewed in [34]). This triggers the activation of signaling cascades that lead to vasodilation, anti-proliferative, anti-inflammatory, and metabolic benefits [34]. The balance between the canonical and non-canonical RAS axes is crucial for maintaining vascular tone and blood pressure homeostasis (Figure 1).

In addition to the systemic, circulating RAS, there are also local/tissue specific RAS in nearly every organ, including the heart, kidney, brain, vasculature, adipose tissue, pancreas, and reproductive organs (reviewed in [35]). These local RAS act in autocrine, paracrine and/or intracrine fashions, independently of the circulating system, and play crucial roles in tissue-specific regulation of growth, repair, fibrosis, metabolism, inflammation, and microcirculatory control. Dysregulation of local RAS has been linked to cardiac diseases, chronic kidney disease, T2D, and neurodegeneration [35].

The RAS can be modulated by ACE inhibitors and angiotensin receptor blockers (ARBs). The enzyme ACE has a dual function: it generates Ang II from Ang I, but it also degrades bradykinin. Bradykinin is a nonapeptide that is generated from kininogen by kallikrein and is widely expressed in vascular and other tissues. It promotes vasodilation, increases vascular permeability, and contributes to inflammatory signaling [36]. Therefore, ACE inhibitors not only reduce Ang II formation but also block bradykinin breakdown, leading to enhanced bradykinin activity. This contributes to the antihypertensive and vasodilatory effects of ACE inhibitors but also explains side effects such as dry cough and angioedema [36]. ARBs, on the other hand, selectively block Ang II binding to AT1R, leaving circulating Ang II intact but unable to exert its actions. Both ACE inhibitors and ARBs are widely used in clinical practice to manage hypertension, chronic kidney disease, and heart failure [36].

## 3. Pharmacological Inhibition of RAS: Implications for Metabolic Aging in Humans

### 3.1. Aging and Metabolism

Advancing age in humans is accompanied by marked changes in key metabolic processes that affect energy regulation, glucose metabolism, fat storage, and muscle maintenance [37]. The metabolic tissues most affected are adipose tissue and skeletal muscle, each undergoing characteristic age-related changes that collectively drive systemic metabolic decline and increase the risk of T2D, cardiovascular disease, frailty, and sarcopenia [37,38]. These complex metabolic changes have been recently reviewed by Khalaf et al. [39].

One of the manifestations of metabolic aging is a regional redistribution of adipose tissue, which typically begins in mid-adulthood (around the fourth to fifth decade) [40], coinciding with the menopausal transition in women [41]. This shift is characterized by increased central fat accumulation, particularly in visceral depots, accompanied by a progressive loss of subcutaneous adipose tissue in the gluteal–femoral regions [40]. These depot-specific alterations in fat storage are largely driven by the reduced lipid-storage capacity of mature adipocytes and the diminished proliferative potential of preadipocytes in the lower body subcutaneous fat [42]. As a result, excessive release of free fatty acid and lipid deposition occurs in visceral depots and non-adipose tissues, such as the skeletal muscle and liver, promoting systemic lipotoxicity and increased production of pro-inflammatory cytokines and chemokines [42]. Over time, these processes contribute to metabolic syndrome, which is characterized by visceral obesity, insulin resistance, dyslipidemia, and hypertension, and is a major risk factor for type 2 diabetes and cardiovascular disease in older adults [43].

Age-related changes in adipose tissue contribute significantly to the development of insulin resistance in skeletal muscle through intramyocellular lipid accumulation, impaired insulin signaling, and sustained chronic low-grade inflammation [37]. At the same time, skeletal muscle undergoes profound structural and functional changes with age that further exacerbate systemic metabolic decline (reviewed in [44]). One of these changes is referred as sarcopenia, the progressive loss of muscle mass and strength, which leads to frailty and reduced physical performance while elevating the risk of morbidity and mortality [44]. Sarcopenia has also important implications for basal metabolic rate (BMR) and overall energy balance. As the largest contributor to BMR, accounting for ~20–30% of total resting energy expenditure [45], skeletal muscle loss during aging contributes to the decline in BMR that typically begins around 60 years of age [46] and further limits mobility, thereby lowering physical activity and total daily energy expenditure [47]. While reduced BMR associated with sarcopenia can indirectly promote adverse metabolic consequences, recent genetic evidence indicates that BMR itself may also influence longevity. In a Mendelian randomization study, Ng and Schooling [35] analyzed sex-specific genetic predictors of BMR in the UK Biobank and found that higher genetically predicted BMR was associated with reduced parental attained age, with a stronger effect observed in women than in men. These findings align with classic theories of aging, such as the “rate-of-living” [48] and “free radical” [49] hypotheses, which propose that increased metabolic activity accelerates aging through oxidative stress. Taken together, these observations suggest that, although through distinct mechanisms, both reduced and elevated BMR can be detrimental to healthy aging, highlighting the importance of maintaining metabolic balance across the lifespan.

At the cellular level, these systemic changes are underpinned by age-related alterations that directly impair metabolic homeostasis, including mitochondrial dysfunction, genome instability, epigenetic alterations, impaired proteome homeostasis, and cellular senescence [37]. Mitochondrial dysfunction in adipose tissue and muscle reduces oxidative phosphorylation efficiency, increases the production of reactive oxygen species (ROS), and limits energy availability, while senescent cells that accumulate in these tissues secrete pro-inflammatory factors that exacerbate insulin resistance and tissue degeneration. Overall, these processes accelerate metabolic decline and link cellular aging to organismal vulnerability to age-related diseases [37].

### 3.2. RAS Blockade and Metabolic Aging

The RAS has emerged as a key modulator of metabolic aging. Chronic activation of the canonical ACE/Ang II/AT1R axis promotes visceral adiposity, insulin resistance, and low-grade inflammation [50], all of which contribute to the development of metabolic syndrome and age-related cardiometabolic decline. A central mechanism involves RAS-driven oxidant production through NADPH oxidases, which results in excess ROS generation and consequent impairment of insulin signaling [51]. However, as reviewed by de Cavanagh et al. [24], dysregulation of the balance between ACE/Ang II/AT1R and ACE2/Ang-(1–7)/Mas receptor axes intersects with several hallmarks of aging, including mitochondrial dysfunction and cellular senescence. Notably, although ACE inhibitors and ARBs have been in clinical use for over 35 years as first-line therapies for hypertension, they also protect target organs, including the kidneys, brain, and heart [52], decrease mortality and morbidity in heart failure patients [52], and slow progression of chronic kidney disease [53]. This broad spectrum of benefits positions RAS blockade as an attractive pharmacological strategy not only for blood pressure control but also for mitigating mechanisms underlying metabolic aging and extending healthspan [24].

In relation to skeletal muscle and impairment of mobility and physical function with aging, clinical studies provide evidence that RAS blockade can attenuate age-related mobility decline in select populations, although the findings are mixed. A randomized landmark trial in older adults with functional impairments found that 20 weeks of perindopril treatment significantly improved walking distance on a six-minute walking test [54]. Similarly, in a large longitudinal observational cohort of older women with hypertension, continuous use of ACE inhibitors over three years was significantly associated with slower age-related decline in muscle strength and walking speed compared to intermittent users or non-users of antihypertensive drugs [55]. A cross-sectional analysis of data from the Health, Aging and Body Composition study further suggested that ACE inhibitors users may preserve greater lower-extremity muscle mass, consistent with a potential muscle-preserving action [56]. Moreover, secondary analyses of the Lifestyle Interventions and Independence for Elders Pilot trial, which tested the effects of chronic exercise on mobility outcomes in older adults at risk for disability, reported greater clinically meaningful gains in walking speed and a battery of short-duration mobility tasks among ACE inhibitor users than non-users, suggesting a possible synergy between RAS blockade and physical activity [57]. However, a recent systematic review and meta-analysis [58], as well as a randomized controlled trial testing the efficacy of perindopril in older adults with sarcopenia [59], did not consistently demonstrate improvements in endurance, muscle strength, or composite physical function scores. These findings suggest that the benefits of RAS blockade on healthspan are context dependent. Studies on *Drosophila* enable genetic and pharmacological dissection of RAS-related pathways related to senescence of muscle tissue and locomotor ability, thereby uncovering conserved mechanisms that cannot be readily resolved in human populations.

## 4. ACE-like Enzymes in *D. melanogaster*: Pharmacological Inhibition and Effects on Metabolic Aging

### 4.1. Functional Roles of ACE-like Enzymes in Drosophila

Despite lacking the canonical mammalian renin-angiotensin system, *D. melanogaster* expresses pharmacologically targetable ACE-like enzymes. Two catalytic orthologs of human ACE have been identified: angiotensin-converting enzyme (Ance) and angiotensin-converting enzyme-related (Acer) [60], which share 45% and 41% amino acid sequence identity with mammalian ACE, respectively, and both retain the canonical HExxH zinc-binding motif and catalytic glutamate characteristic of ACE family peptidases (see [61]). However, Ance closely resembles ACE in its ability to convert angiotensin I to angiotensin II, hydrolyze bradykinin, and bind human ACE-specific inhibitors such as captopril and lisinopril [62]. Conversely, Acer shows substrate specificity for a distinct set of bioactive peptides and does not hydrolyze angiotensin I, highlighting functional divergence between the two enzymes [63].

Developmental studies have demonstrated that Ance plays a critical functional role during metamorphosis [60]. Its enzymatic activity contributes to stage-specific peptide metabolism, coordinating both cuticular remodeling and the development of internal organ systems [60]. During this transition, energy reserves accumulated during the larval stage are strategically redistributed to support pupal development while also provisioning for the future reproductive and survival needs of the new adult [64]. Because Ance peptidase activity peaks during the critical period of post-metamorphic energy reallocation, it is likely to influence how energy is partitioned and set the stage for the future trajectory of metabolic aging. This idea is supported by evidence that Ance impacts both reproduction [65] and lifespan regulation [66]. Furthermore, pharmacological inhibition of Ance with lisinopril extends lifespan in Drosophila, an effect that is abolished by muscle-specific *Ance* knockdown [66]. This implicates muscle-expressed Ance in the age-associated physical decline in *D. melanogaster* and reinforces reports linking Ance activity to locomotor performance [61]. These findings are also consistent with observations from human cohort studies [67,68] and muscle-targeted genetic interventions in Drosophila [69,70], both highlighting skeletal muscle as a central regulator of lifespan and age-related pathologies in non-muscle tissues. In humans, physical inactivity drives metabolic deficits characterized by disruptions in skeletal muscle and adipose tissue that are often irreversible in older adults [71,72]. Similarly, prolonged inactivity shortens lifespan and impairs muscle homeostasis in Drosophila [73], underscoring conserved mechanisms across species. Taken together, these findings position Ance as a central modulator of both healthspan and lifespan, exerting lasting effects through developmental programming and adult muscle-specific functions also implicated in human aging.

In contrast to Ance, Acer is considered the Drosophila ortholog of mammalian ACE2 and has been implicated in both Drosophila cardiac physiology and lifespan regulation [74]. Functional studies with *Acer* null mutants have further revealed that Acer is expressed in the adult fat body, where it regulates sleep and metabolism in both males and females in a nutrient-dependent manner [75].

### 4.2. ACE Inhibitors, Metabolism, and Aging in D. melanogaster

A growing body of research strongly suggests that ACE inhibitors, particularly lisinopril, modulate lifespan, metabolism, and mitochondrial function of *D. melanogaster*. Gabrawy et al. [66] examined lifespan and physical resilience during aging in males from three genetically diverse wild-type lines of the Drosophila Genetic Reference Panel (DGRP) [76,77] treated with lisinopril compared to untreated controls. They found that lisinopril extended lifespan in all three lines, although the magnitude of benefit varied across lines. On the other hand, lisinopril improved age-impaired physical performance traits, such as climbing speed, endurance, and strength, in a genotype-specific manner. In responsive lines, lisinopril reduced age-associated protein aggregation in skeletal muscle, and muscle-specific knockdown of *Ance* abolished its lifespan benefits. Furthermore, transcriptome analyses revealed that the drug’s effects on gene expression were both age- and genotype-dependent, particularly involving the stress and immune response pathways. Together, these findings indicate that the mechanisms by which lisinopril promotes longevity are at least partly distinct from those that preserve functional healthspan and underscore genetic background as a key determinant of ACE inhibitor efficacy. This is particularly relevant for human populations, where genetic information may be required to guide personalized strategies for lisinopril-mediated healthspan extension.

In addition to their effects on longevity, recent studies have shown that ACE inhibitors and ARBs reduce neuronal cell death and enhance memory in Drosophila models of Alzheimer’s disease (reviewed in [78]). Thomas et al. [79] reported that lisinopril significantly improved memory and motor function in male flies overexpressing human amyloid precursor protein and β-secretase, which is the enzyme involved in the first step leading to the production of the amyloid-beta peptide [80]. These findings confirm previous work showing neuroprotective effects of the ACE inhibitor captopril and the ARB losartan in Drosophila amyloid-based models [81]. However, neither drug rescued deficits in flies expressing human tau, indicating pathway-specific efficacy. Importantly, these beneficial effects occurred independently of changes in amyloid pathology.

**Figure 2:**
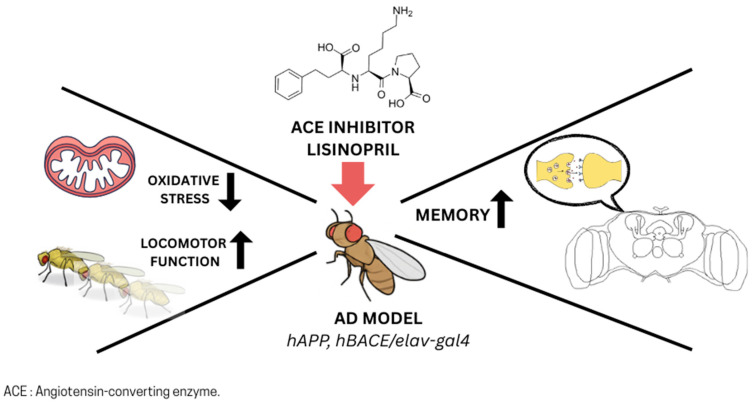
Neuroprotective and functional effects of ACE inhibitors in *Drosophila* Alzheimer’s disease models. Treatment with ACE inhibitors (e.g., lisinopril, captopril) reduces neuronal cell death and improves memory in flies overexpressing human amyloid precursor protein and β-secretase in the neuron system. Lisinopril also enhances locomotor function, associated with reduced oxidative stress and improved mitochondrial dynamics in muscle tissue.

The study by Thomas et al. [79] also revealed that the locomotor benefits of lisinopril were accompanied by a reduction in ROS in muscle tissue, implicating decreased oxidative stress as a contributor to improved physical function. This observation is consistent with other evidence that lisinopril modulates ROS levels and mitochondrial dynamics in *D. melanogaster* (see below) [82]. Because both oxidative stress and mitochondrial dysfunction are hallmarks of aging, and are well documented in mammalian tissues with RAS dysregulation [83,84], these observations support a conserved role for ACE inhibition in mitigating age-related decline through redox and mitochondrial pathways (Figure 2).

**Figure 3:**
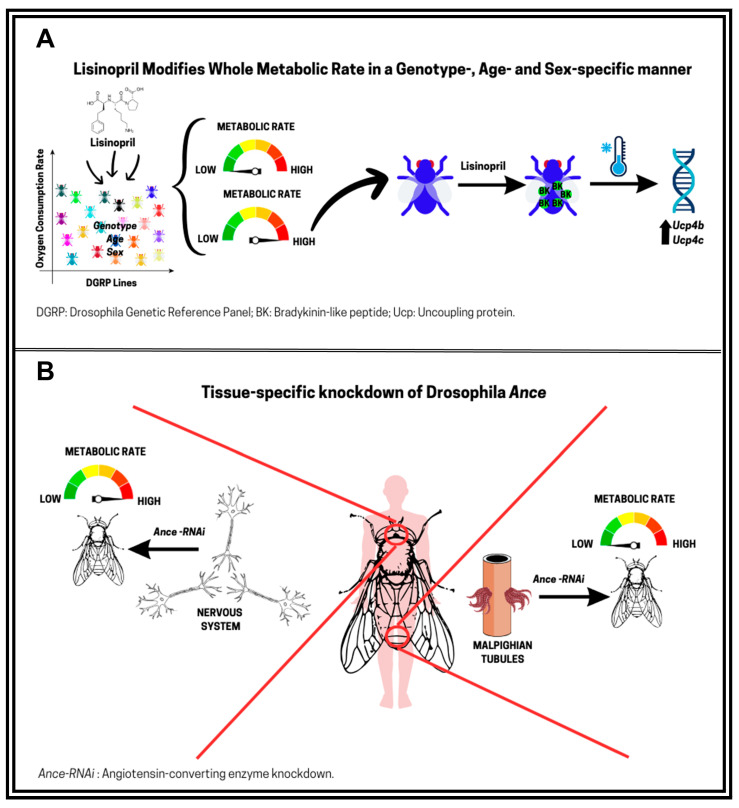
Lisinopril modulates metabolic rate in a genotype-, age-, and sex-specific manner through conserved and tissue-specific mechanisms in *D. melanogaster.* (**A**) Exposure to lisinopril alters whole-body metabolic rate in Drosophila Genetic Reference Panel (DGRP) lines, with responses varying by genotype, age, and sex. Increased metabolic rate is associated with elevated cold resistance and upregulation of uncoupling proteins (*Ucp4b* and *Ucp4c*) in the heads of male flies, suggesting a thermogenic mechanism potentially mediated by bradykinin-like peptides. (**B**) Tissue-specific RNAi knockdown of *Ance*, the Drosophila ortholog of ACE, in the nervous system and Malpighian (renal) tubules results in opposite effects on metabolic rate. The metabolic effects of lisinopril are abolished in *Ance*-deficient tissues in a sex- and age-dependent manner. These findings underscore the importance of tissue context in ACE inhibitor action and support the utility of Drosophila for dissecting gene x drug interactions.

To further investigate the metabolic effects of lisinopril in *D. melanogaster*, Ederer et al. [82] assessed mitochondrial function, mitochondria content, and ROS levels in thoraces (mainly muscle-enriched) of young (one-week-old) and middle-aged (three-week-old) males from the same three DGRP lines used by Gabrawy et al. [66]. Lisinopril reduced thoracic ROS levels and mitochondrial respiration in young flies while increasing mitochondrial content in middle-aged flies in a genotype-specific manner [82]. Building on this work, Vecchie’ et al. [85] subsequently assessed whole-body metabolic rate across 22 genetically diverse DGRP lines. Male and female flies were treated with lisinopril for one or five weeks, revealing significant inter-individual variation in metabolic response. While some lines exhibited increased metabolic rates, others showed reductions, with both age and sex exerting strong influences (Figure 3). Treatment with the ARB losartan produced effects similar to lisinopril, suggesting that metabolic rate may be modulated by inhibition of an ancestral Ang II-like pathway. However, losartan did not always mimic the effects of lisinopril; for example, it did not influence metabolic rate in young DGRP-367 and old DGRP-808 flies [85]. This divergence suggests that lisinopril may also act through alternative mechanisms, such as inhibition of bradykinin-like peptide degradation. Untargeted metabolomics added further insights by showing that lisinopril perturbs thoracic metabolic networks, particularly glycolysis, mevalonate metabolism, and glycogen degradation [82]. Notably, lisinopril decreased survival under nutrient starvation in an age- and genotype-dependent manner, paralleling the observed metabolic alterations [82]. Altogether, these results suggest that lisinopril can influence nutrient-dependent fitness traits in *D. melanogaster* through mitochondrial mechanisms.

### 4.3. Effects of Lisinopril on Thermoregulation in D. melanogaster

Because metabolic rate and energy homeostasis are closely linked to temperature regulation, the effects of lisinopril were also assessed on chill-coma recovery time, which was used as an indicator of cold tolerance [85]. In young DGRP-367 and old DGRP-808 flies, lisinopril treatment improved cold tolerance and enhanced metabolic rate. The expression of the uncoupling protein genes *Ucp4b* and *Ucp4c* in these flies was also markedly increased in the head, but not in thoracic or abdominal tissues, suggesting that lisinopril may stimulate thermogenesis through UCP activation in the head fat cells and/or brain of flies. Furthermore, tissue-specific knockdown of *Ance* in the nervous system and Malpighian (renal) tubules affected metabolic rate in opposite directions, highlighting complex, tissue-dependent roles for Ance inhibition [85]. Overall, these results indicate that the systemic effects of lisinopril on Drosophila metabolism are context-dependent and likely reflect the integration of tissue-specific processes (Figure 3). Potential mechanisms involved in the effects of lisinopril on Drosophila metabolic rate include reduced energy demand from Na^+^/K^+^-ATPase activity and sodium reabsorption in renal tissues, improved fluid–electrolyte balance, and modulation of sympathetic nervous system activity, all of which may converge to influence thermogenic and hormonal regulation of metabolism.

Comparable mechanisms have been described in mammals. It is well established that cold exposure activates the sympathetic nervous system, which triggers catecholamine release that, in turn, stimulates brown adipose tissue thermogenesis [86] and peripheral vasoconstriction [87] to preserve heat. In this context, recent work in male mice showed that ACE inhibitors enhanced *UCP1* expression and thermogenesis in brown adipose tissue and elevated body temperature via beta-2 adrenergic receptor signaling by reducing bradykinin degradation [88]. Thermoregulatory mechanisms decline with aging, with individuals over 60 years often displaying lower body temperature [89] and impaired brown adipose tissue activity [90]. Studies in mice have shown that compromised thermogenesis contributes to hypothalamic neurodegeneration and impaired feedback control of body temperature [85]. These observations, together with findings in flies, motivate additional studies in Drosophila to dissect the mechanisms by which lisinopril influences thermoregulation. Such work could provide mechanistic insights that ultimately inform whether lisinopril has therapeutic potential to improve thermoregulatory capacity and help prevent or delay neurological or neurodegenerative disorders in the elderly.

## 5. Conclusions

Findings from evolutionary theory, molecular hallmarks of aging, and pharmacological studies have established metabolic regulation as a central determinant of aging trajectories and age-related disease, positioning it as a promising target for interventions aimed at extending healthspan. Within this framework, inhibition of RAS has emerged as a plausible strategy, with evidence from mammalian models and *D. melanogaster* pointing to beneficial effects on lifespan, healthspan, and the ability to maintain or restore metabolic homeostasis under stress. Studies in Drosophila have also revealed that the effects of ACE inhibitors are strongly influenced by age, sex, and genetic background, and that the mechanisms extending lifespan may differ from those preserving healthspan. These findings underscore the need for additional studies in Drosophila to dissect evolutionary trade-offs, define the tissue-specific actions of ACE-like enzymes, and identify the molecular pathways driving the observed benefits, while determining the extent to which these mechanisms are conserved in mammals, including humans. Integrating Drosophila research with rodent and human cohort studies will be essential to establish whether pharmacological blockade of RAS can be leveraged to promote healthy aging.

## Data Availability

No new data were created or analyzed in this study.
